# Acanthosis nigricans in type 2 diabetes: prevalence, correlates and potential as a simple clinical screening tool - a cross-sectional study in the Caribbean

**DOI:** 10.1186/1758-5996-6-77

**Published:** 2014-07-09

**Authors:** Sarasvati Bahadursingh, Catherine Mungalsingh, Terence Seemungal, Surujpal Teelucksingh

**Affiliations:** 1 Department of Clinical Medical Sciences, The University of the West Indies, St. Augustine, Trinidad and Tobago W.I

**Keywords:** Acanthosis nigricans, Type 2 diabetes mellitus, Prevalence, Obesity, Screening, Risk factor, Age, Sex, Ethnicity, BMI, Waist circumference

## Abstract

**Background:**

This study aimed to evaluate the role of acanthosis nigricans (AN) as a marker of Type 2 Diabetes Mellitus (T2DM) by studying its prevalence and relationship with age, ethnicity, anthropometry and other risk factors for T2DM in the Trinidadian population.

**Methods:**

311 successive adult patients with T2DM were recruited at diabetic clinics and inpatient wards across Trinidad. The presence, severity and texture of AN at the neck were assessed. Demographic, clinical and anthropometric characteristics were also measured, and logistic regression was used to model their relationship with presence of AN.

**Results:**

The mean (SD) age was 58.1 years (12.6). 55.6% were female. 61.1% were East Indian, 24.4% African and 14.5% mixed ethnicity. The mean (SD) BMI was 27.3 kg/m^2^ (6.0) and the mean (SD) waist circumference was 96.7 cm (14.2). Prevalence of AN was 52.7% (95% CI 47.2, 58.3).

There was a greater odds of AN among diabetic patients who were: younger (p < 0.001); female (OR 1.67; 95% CI 1.06, 2.62); or East Indian rather than African (0.45; 0.26, 0.77) or mixed (0.43; 0.22, 0.84) descendents. There was a greater age-, sex- and ethnicity-adjusted odds of AN among those: overweight (3.98; 2.10, 7.55) or obese (8.31; 3.84, 18.00) versus normal BMI; centrally obese (4.72; 2.65, 8.43); with history of hypertension (2.19; 1.27, 3.79) or history of hypercholesterolemia (1.72; 1.02, 2.90), but there was no evidence of this demographic-adjusted association (p > 0.4) between AN and history of previous MI or CVA, family history of T2DM, T2DM treatment regimen, duration of T2DM or random blood glucose.

On further multivariable analysis, only age, sex, ethnicity, BMI and waist circumference were independently associated with AN (p < 0.05) and the effect of BMI varied with ethnicity.

**Conclusions:**

There was a high prevalence of AN both overall and across age, sex and ethnic groups of diabetic patients. AN exhibited much potential as a valuable addition to T2DM risk assessment in the Trinidadian and similar settings.

## Background

Trinidad and Tobago is a twin-island unitary republican state in the Southern Caribbean with a population of 1.3 million inhabitants derived mostly of descendants from peoples of India (35%) and West Africa (34%) with a sprinkling of others of Chinese, Syrian/Lebanese and Caucasoid stock. Arising out of this background is an expanding group of mixed ethnicity who currently accounts for approximately 23% of the population [1].

As early as the 1960’s, a national survey of more than 20,000 individuals revealed an adult prevalence of Type 2 diabetes mellitus (T2DM) of 3.5%, already one of the highest in the Hemisphere, and identified a higher prevalence among Indian descendants [2]. Twenty years later, the St James Survey, a longitudinal study in an urban setting, confirmed this persistently increased risk for diabetes among East Indians, about twice that for Africans. Interestingly people of mixed ancestry were identified in that study to exhibit low risk for both diabetes and its associated complication, ischaemic heart disease [3].

More recently data from the Trinidad and Tobago Chronic Non-Communicable Disease Risk Factor (Pan American STEPS) Survey showed a prevalence of T2DM of 20% among individuals between 15–64 years of age [4]. Against this background, it is therefore not surprising that T2DM and its complications account for considerable use of resources, bed space and clinical load [5].

Given this burden of disease, clinicians require sensitive, safe and inexpensive screening tests so that interventions can be instituted earlier. In some populations, acanthosis nigricans (AN) has been recommended as a suitable tool for such screening [6], as its association with T2DM, insulin resistance and obesity is well-established [7,8]. However, studies have reported that the prevalence of AN varies greatly in different ethnic groups [9] and any recommendation for its use must be relevant to the population for which this is intended. Thus Grandhe et al. report that up to two-thirds of type 2 diabetic patients from North India will display this sign [10] whereas a study from Nigeria showed that less than 20% will so do [11]. In general, darker races are more likely to exhibit this sign than are Whites [9].

This study aimed to evaluate the role of AN as a marker of T2DM by studying its prevalence and relationship with age, ethnicity, anthropometry and other risk factors for T2DM in the Trinidadian population.

## Methods

This is a cross-sectional study. Three hundred and eleven (311) successive subjects were recruited at dedicated diabetic outpatient clinics and the general medical and surgical inpatient wards at the Port of Spain General Hospital, San Fernando General Hospital and Eric Williams Medical Sciences Complex in Trinidad. These hospitals are the three main public hospitals and together serve a national population of 1.3 million. Approval was obtained from the ethics committees of participating hospitals and of the University of the West Indies, and research was in compliance with the Helsinki Declaration.

Included in this study were individuals 18 years of age and older with T2DM (according to the World Health Organisation (WHO) definition [12]) and who were able to give informed consent. Patients were excluded if they had other endocrine or systemic diseases known to result in AN. More specifically, patients with a history of, being investigated for, or diagnosed with one or more of the following were excluded: internal malignancy (gastrointestinal); autoimmune conditions such as systemic lupus erythematosus, scleroderma, Sjogren’s syndrome and Hashimoto’s thyroiditis. Also excluded from the study were subjects with a history of intake of drugs which can cause AN (nicotinic acid, oral contraceptives, topical fusidic acid, methyltestosterone and triazinate) [8,9] as well as subjects who were unable to give a cohesive history, had a high alcohol intake (>14 units/week for women and >21 units/week for men), used illicit drugs, were less than 18 years of age or who did not have T2DM.

The main outcome measure was AN. Subjects were examined for the presence of AN at the neck, and its severity and texture were graded using standard scales as described by Burke et al. [13]. Severity was graded from 0 to 4 as follows: grade 0 - not visible on close inspection; grade 1 - clearly present on close visual inspection; grade 2 - limited to base of the skull but does not extend to the lateral margins of the neck; grade 3 - extending to the lateral margins of the neck but not visible from the front; and grade 4: extending to the anterior neck. The texture of AN at the neck was graded from 0 to 3 as follows: Grade 0 - smooth to touch, no differentiation from normal skin to palpation; Grade 1- rough to touch, clearly differentiated from normal skin; Grade 2 - coarseness can be observed visually, portions of the skin clearly raised above other areas; and Grade 3 - extremely coarse, “hills and valleys” observable on visual examination. The presence of AN at other typical sites, including knuckles, elbows, axillae and knees, was also documented. Those patients with localized hyperpigmentation without skin thickening were not considered to have AN [13]. AN was assessed by a single trained observer.

Demographic and clinical characteristics, including anthropometric measurements, were also taken. An administered questionnaire was used to collect data on: age; sex; self-reported ethnicity; previous history of hypercholesterolemia, hypertension, myocardial infarction (MI) and/or cerebrovascular accident (CVA); family history of T2DM; duration of T2DM; and treatment with oral hypoglycaemics, insulin or both. The absence of the exclusion criteria mentioned previously was also ascertained. The most recent random blood glucose (RBG) level was obtained from medical records as a surrogate for blood glucose control in the absence of consistently available HbA1c. Anthropometric measurements taken included the weight and height of all subjects. These were measured according to usual clinical practice, and body mass index (BMI) was calculated as weight in kilograms divided by height in metres squared. Waist circumference was measured midway between the lower margin of the ribcage and the superior rim of the ilium using a flexible tape measure.

### Statistical analysis

Data were analysed using Stata Version 13.0. All statistical tests were interpreted at the 5% level of significance. Population characteristics were summarised. The distribution of AN both overall and by each population characteristic was examined. The effect of each explanatory variable on the odds of AN was summarised using odds ratios (ORs) with 95% confidence intervals (CIs) that were adjusted for the confounding effect of demographic characteristics of age, sex and ethnicity in a logistic regression model. The likelihood ratio test (LRT) was used to test the age-, sex- and ethnicity-adjusted association between AN and each explanatory variable. Age, BMI and waist circumference were categorised using clinically relevant cut-points for data summary, measures of effect and hypothesis testing. Age was adjusted for in continuous form.

Multivariable logistic regression modelling was used to further explore confounding by other explanatory variables. All measured variables were included in the initial model and backward elimination of terms was performed: terms with the largest p values on the Wald test were eliminated stepwise until all terms had p < 0.05. Subsequently, interactions of all retained terms with age, sex and ethnicity were investigated and retained if p < 0.05. All continuous variables were modelled in original form. This final model determined was later refitted with BMI in categorical form to further explore and interpret the interaction detected.

Characteristics found to be independently associated with the presence AN were further evaluated for a simple linear trend with increasing severity of AN – for descriptive interest. The chi-squared test for trend was used to evaluate evidence of a trend in the proportion of participants with a binary characteristic across increasing grades of AN severity; each ethnic group was treated as a binary characteristic. The ANOVA trend test was used to assess linear trends in the mean of continuous variables across increasing severity of AN. Regression coefficients were subsequently evaluated and tested in linear and logistic regression models for continuous and categorical variables respectively. The Cuzick [14] nonparametric extension of the Wilcoxon rank-sum test [14] was used to test for a trend in the average texture of AN across increasing grade of AN severity.

An estimated sample size of 272 participants was required to estimate the prevalence of AN among patients with T2DM with a total confidence interval width of 0.10, confidence level of 90%, and an expected proportion of AN of 0.50. The estimated prevalence of AN in T2DM varied widely in the literature from 36% in one study of a diverse ethnic population [15] to 63% in a population of North Indians [10]. An expected proportion of AN of 0.5 therefore chosen since it corresponded to the largest estimated sample size.

## Results

### Sample characteristics

The study sample comprised 311 patients with T2DM. Their demographic and clinical characteristics are summarised in Table 1. Their mean age was 58.1 years (SD 12.6; range 25–99 years). 55.6% were female. 61.1% were East Indian, 24.4% African and 14.5% mixed ethnicity. The mean BMI was 27.3 kg/m^2^ (SD 6.0) and the mean waist circumference was 96.7 cm (SD 14.2).

**Table 1:** Acanthosis Nigricans (AN): prevalence, and age-, sex- and ethnicity-adjusted associations with demographic and clinical characteristics

**Characteristic**	**Subgroup**	**Overall frequency of characteristic**		**Prevalence of AN in subgroup**		Age^*^-, sex- & ethnicity-adjusted		
		**N**	**(%)**	**N**	**(%)**	**OR (95% CI)**^**†**^		**p value**^**‡**^
Age (years)^†^	25 - 39	28	(9.0)	21	(75.0)	5.84	(2.11, 16.18)	
	40 - 49	42	(13.5)	30	(71.4)	4.87	(2.04, 11.60)	
	50 - 59	94	(30.2)	56	(59.6)	2.87	(1.44, 5.72)	
	60 - 69	91	(29.3)	38	(41.8)	1.40	(0.70, 2.79)	
	≥70	56	(18.0)	19	(33.9)	1		<0.001
	All [*mean(SD)*]	[58.1	(12.6)]					
Sex^†^	Males	138	(44.4)	63	(45.7)	1		
	Females	173	(55.6)	101	(58.4)	1.67	(1.06, 2.62)	0.026
Ethnicity^†^	East Indian	190	(61.1)	115	(60.5)	1		
	African	76	(24.4)	31	(40.8)	0.45	(0.26, 0.77)	
	Mixed	45	(14.5)	18	(40.0)	0.43	(0.22, 0.84)	0.003
Body Mass Index	Underweight	6	(2.2)	0	(0.0)			
(kg/m^2^)^§, ¶^	Normal	106	(38.5)	32	(30.2)	1		
	Overweight	94	(34.2)	58	(61.7)	3.98	(2.10, 7.55)	
	Obese	69	(25.1)	53	(76.8)	8.31	(3.84, 18.00)	<0.001
	All [*mean(SD)*]	27.3	(6.0)					
Waist Circumference	Normal	148	(50.5)	54	(36.5)			
(cm)^§, **^	Central obesity	145	(49.5)	99	(68.3)	4.72	(2.65, 8.43)	<0.001
	All [*mean(SD)*]	96.7	(14.2)					
Hypertension	No	104	(33.4)	49	(47.1)	1		
	Yes	207	(66.6)	115	(55.6)	2.19	(1.27, 3.79)	0.004
Hypercholesterolemia	No	121	(38.9)	56	(46.3)	1		
	Yes	190	(61.1)	108	(56.8)	1.72	(1.02, 2.90)	0.040
Family history of T2DM^§^	No	54	(17.4)	22	(40.7)	1		
	Yes	256	(82.6)	141	(55.1)	1.3	(0.68, 2.48)	0.420
CVA or MI	No	233	(74.9)	122	(52.4)	1		
	Yes	78	(25.1)	42	(53.8)	1.26	(0.72, 2.22)	0.413
Diabetes Treatment	Oral	192	(61.7)	101	(52.6)			
	Oral & Insulin	28	(9.0)	11	(39.3)	0.63	(0.26, 1.54)	
	Insulin	91	(29.3)	52	(57.1)	1.14	(0.67, 1.96)	0.467
RBG (mg/dL)^§^	All [*med(IQR)*]	[145	(84)]			1.00	(0.998, 1.004)	0.584
Duration of T2DM (yrs)^§^	All [*med(IQR)*]	[10	(16)]			1.00	(0.98, 1.03)	0.847
**Overall**		311	(100)	164	(52.7)			

*Abbreviations*: *AN* Acanthosis Nigricans, *OR* Odds Ratio, *CI* Confidence Interval, *SD* Standard Deviation, *BMI* Body Mass Index, *T2DM* Type 2 Diabetes Mellitus, *CVA* Cerebrovascular accident, *MI* Myocardial Infarction, *RBG* Random Blood Glucose, *med* median, *IQR* Inter-quartile range. ^*^age adjusted for as a continuous variable; ^†^Odds Ratios for variables age, sex and ethnicity are univariable analyses; ^‡^p value for age-, sex- and ethnicity-adjusted association with AN from likelihood ratio test; ^§^36, 18, 1, 14, 1 missing observations for BMI, waist circumference, family history of T2DM, RBS and duration of T2DM respectively. No missing observations for other variables. ^¶^ BMI (kg/m^2^): Underweight: <18.50; Normal: 18.50-24.99; Overweight: 25.00-29.99; Obese ≥30.00 [16]. ^**^Central Obesity: waist circumference >102 cm in men and >88 cm in women [17].

### Prevalence of AN

The prevalence of AN among all study participants was 52.7% (95% CI 47.2, 58.3). Table 1 shows the estimated prevalence of AN in different subgroups. Of note, 58.4% of females compared to 45.7% of males had AN. The prevalence of AN decreased from 75.0% in the youngest age group (25–39 years) to 33.9% in the oldest age group (≥70 years). 60.5% of participants of East Indian descent had AN as compared to 40.8% and 40.0% of participants of Africans and mixed descent respectively. The prevalence of AN among normal-BMI participants was 30.2% as compared to 61.7% and 76.8% among overweight and obese participants respectively and 68.3% of those with central obesity had AN compared to 36.5% of those without.

### Age-, sex- and ethnicity-adjusted associations with AN

Table 1 further compares the prevalence (via odds ratios) of AN in different demographic and clinical subgroups. There was a greater odds of AN among those who were: younger (p < 0.001); female (OR 1.67; 95% CI 1.06, 2.62) and of East Indian descent rather than of African (OR 0.45; 95% CI 0.26, 0.77) or mixed (OR 0.43; 95% CI 0.22, 0.84) descent. After adjusting for the confounding effect of demographic features, there was a greater age- sex- and ethnicity-adjusted odds of AN among those who were: overweight (OR 3.98; 95% CI 2.10, 7.55) or obese (OR 8.31; 95% CI 3.84, 18.00) compared to participants of normal BMI; had central obesity (OR 4.72; 95% CI 2.65, 8.43); had a history of hypertension (OR 2.19; 95% CI 1.27, 3.79) or a history of hypercholesterolemia (OR 1.72; 95% CI 1.02, 2.90).

There was no evidence (p > 0.40) of an age-, sex- and ethnicity- adjusted association between AN and a history of previous MI or CVA, a family history of T2DM, T2DM treatment regimen, the duration of T2DM or the RBG (Table 1).

### Multivariable analysis

Backward elimination of variables was performed, and apart from the demographic characteristics of age, sex and ethnicity, only BMI and waist circumference remained significantly associated with AN (p < 0.05). A history of hypercholesterolemia or hypertension were no longer associated with AN after also adjusting for the effect of anthropometry. Remaining characteristics, which were not found to be associated with AN in previous analysis, remained that way and were eliminated from the model. Further investigation for interactions showed that the effect of BMI varied by ethnicity. There was no evidence of an interaction (p > 0.05) of any of the other variables in the model with either age, sex or ethnicity.

This final multivariable model is shown in Table 2. Holding other variables in the model constant, females had a 2.33 greater odds of AN than males (95% CI 1.27 - 4.27). A 10 year decrease in age and 10 cm increase in waist circumference was associated with a 1.87 (95% CI 1.41 - 2.48) and 1.40 (95% CI 0.99 - 1.99) greater odds of AN respectively. Among East Indians, overweight and obese diabetic patients had a 5.51 (95% CI 2.34 - 12.98) and 12.27 (95% CI 2.79 - 53.97) greater adjusted odds of AN respectively. However, among Africans and mixed participants, there was insufficient evidence of an association between BMI and AN. Among obese diabetic patients, East Indians had a greater odds of AN than those of African (OR 0.08; 95% CI 0.01 - 0.45) or mixed (OR 0.14; 95% CI 0.02 - 0.79) descent. In contrast, among normal-weight diabetic patients there was no evidence that the adjusted odds of AN in different ethnic groups was different; it was noted, however, that normal-weight Africans had a 1.7 times greater adjusted odds of AN than normal-weight East Indians, but there was insufficient evidence that this association was not due to chance (p = 0.363).

**Table 2:** Demographic and clinical characteristics independently associated with presence of Acanthosis Nigricans (AN)

**Characteristic**			OR^*^	**(95% CI**^**†**^**)**	**p value**^**‡**^
**Age (years)**^**§**^			0.94	(0.91, 0.97)	<0.001
**Sex**^**§**^		*Males*	1		
		*Females*	2.33	(1.27, 4.27)	0.006
**Ethnicity**^¶^	x Normal BMI^**^	*East Indian*	1		
		*African*	1.70	(0.54, 5.33)	0.363
		*Mixed*	0.68	(0.18, 2.53)	0.570
	x Overweight^**^	*East Indian*	1		
		*African*	0.11	(0.03, 0.41)	0.001
		*Mixed*	0.44	(0.10, 1.83)	0.258
	x Obese^**^	*East Indian*	1		
		*African*	0.08	(0.01, 0.45)	0.004
		*Mixed*	0.14	(0.02, 0.79)	0.027
**Body Mass Index (BMI)**^¶^	**x East Indian**	Normal BMI^**^	1		
		Overweight^**^	5.51	(2.34, 12.98)	<0.001
		Obese^**^	12.27	(2.79, 53.97)	0.001
	**x African**	Normal BMI^**^	1		
		Overweight^**^	0.35	(0.08, 1.64)	0.183
		Obese^**^	0.59	(0.11, 3.12)	0.534
	**x Mixed Ethnicity**	Normal BMI^**^	1		
		Overweight^**^	3.51	(0.60, 20.45)	0.162
		Obese^**^	2.44	(0.37, 16.06)	0.352
**Waist Circumference (cm)**^**§**^			1.03	(1.00, 1.07)	0.060

^*^OR: Odds Ratio of AN adjusted for other variables in the model including age, sex, ethnicity, BMI and waist circumference; ^†^CI: Confidence Interval for adjusted OR; ^‡^p value from Wald test. ^§^Parameter estimates from final multivariable model ^¶^Parameter estimates from final model refitted with BMI as categorical, to illustrate the interaction between BMI and ethnicity. ^**^Body Mass Index (BMI) (kg/m^2^): Underweight: <18.50; Normal: 18.50-24.99; Overweight: 25.00-29.99; Obese ≥30.00 [16].

### Severity and texture of AN

The severity and texture of AN were graded separately. Table 3 summarises the proportion of participants with AN (n = 164) that fell into each of four categories of increasing severity of AN. In general, fewer diabetic patients had a more severe grade of AN: 39.6% were grade 1 compared to 11.6% who were graded as most severe (Grade 4). Participants with AN also fell into one of three grades of increasingly rougher texture. The texture of most AN was grade 1 (83.5%), 12.2% were grade 2 while only 4.3% fell into the highest category (Grade 3). Table 3 summarises the average texture of AN among participants with each grade of AN severity. More severe AN of the neck was associated with a rougher texture of AN of the neck (p < 0.001).

**Table 3:** Variation in the texture and presence of Acanthosis Nigricans (AN) at other sites by its severity

**Severity of AN at the neck**			**Texture of AN at the neck: average grade (SD)**	Proportion with AN at other sites^*^	
	**N**	**(%)**		**N**	**(%)**
*Grade 0 (not present)*	*146*		*-*	*15*	*(10.3)*
Grade I	65	(39.6)	1.00 (0)	35	(53.9)
Grade II	37	(22.6)	1.11 (0.31)	25	(67.6)
Grade III	43	(26.2)	1.28 (0.45)	28	(65.1)
Grade IV	19	(11.6)	1.95 (0.91)	19	(100.0)
Grades I - IV	164	(100)	1.21 (0.50)	122	(39.4)

^*^One missing value for the presence of AN at other sites of the body.

### Linear trends by severity of AN

Characteristics associated with the presence of AN were re-evaluated for a linear trend with increasing severity of AN. It was found that each grade increase in severity of AN was associated linearly with a 2.8 year decrease in age (p = 0.001), 1.8 kg/m^2^ increase in BMI (p < 0.001), a 4.5 cm increase in waist circumference (p < 0.001), a 1.46 times greater odds of being of East Indian descent (95% CI 1.04, 2.06; p = 0.028) and a 0.65 times lower odds of being of African descent (95% CI 0.43, 0.98; p = 0.041). For mixed participants, there was no evidence of a linear trend (OR 0.85; 95% CI 0.53, 1.38; p = 0.515) but the proportion of mixed participants did vary by AN severity (p = 0.045). The proportion of: females, those with a history of hypertension and those with a history of hypercholesterolemia did not vary by severity of AN (p > 0.70) even though persons with these characteristics were overall more likely to have AN (as seen previously).

### AN at other sites of the body

Among all study participants, 107 (34.5%) had AN both at the neck and at other sites, 15 (4.8%) had AN at other sites only, 57 (18.4%) had AN at the neck only and the remaining 131 (42.3%) did not have AN (one missing value for AN at other sites). Therefore, AN was documented at other sites of the body (axillae, elbows, knuckles and/or knees) in 122 (39.4%) participants. Compared to those with AN at the neck, those without AN at the neck had a much lower odds (OR 0.06; 95% CI 0.03, 0.11) of manifesting AN at other sites. As illustrated in Table 3, there was evidence that increasing severity of AN at the neck, was associated with an increasing proportion of diabetic patients with AN at other sites (p = 0.001). Only 3 participants (1.0%) had previously heard of AN.

## Discussion

In Trinidad, screening for T2DM is opportunistic: in clinical settings, individuals are assessed for identifiable risk factors and those subjectively deemed high-risk are recommended for formal screening [18]. Currently, however, more than 50% of diabetic patients remain undiagnosed [18] and among those within reach of opportunistic screening, a major contributor to non-detection is the non-exhaustive collection of risk factors used to determine risk – it can exclude diabetic patients in whom traditional risk assessment is less convincing.

To address this issue, adjuncts to the current set of risk factors are needed but they must be carefully selected. New adjuncts should be not only sensitive, but should improve the sensitivity of the already-in-use pool of risk factors – they should broaden and strengthen the definition of a high-risk individual. Since timely risk assessment is vital in busy clinical settings, adjuncts must be efficiently and safely implementable. Lastly, a specific adjunct will limit the number of individuals wrongly classified as high-risk. Here, we will explore the potential of AN as an adjunct to T2DM risk assessment prior to formal T2DM screening in the Trinidadian population. We will explore the prevalence of AN among diabetic patients, the potential of AN to improve detection of T2DM and the innate qualities of the test that make it suitable.

AN is “a symmetric eruption characterized by hyperpigmented, velvety cutaneous thickening that can occur on any part of the body” [8]. It is considered a cutaneous marker of hyperinsulinemia and insulin resistance [7,19] and has been established as a risk factor for T2DM [6,20,21]. AN has been formally recommended by the ADA as part of the T2DM risk assessment criteria for children and adolescents [22]. However, among adults, the implementation of AN in this capacity has been limited by infrequent manifestation in some populations [9].

In this Trinidad-based study, a relatively high manifestation of AN among diabetic patients was found - as many as 1 in 2 diabetic patients had AN. Of note, AN was 20% more likely to manifest in diabetic patients of East Indian than African or mixed origin – possibly due to a higher prevalence of ectopic fat and associated insulin resistance in the former [23]. However, the prevalence in all of these dominant ethnic groups was quite high: 2 in 5 diabetic patients of either African or mixed origin and 3 in 5 of East Indian origin demonstrated AN. This high prevalence of AN - both overall and among the major Trinidadian ethnic groups - strongly supports its implementation as a sensitive marker of T2DM in the unique ethnic population of Trinidad.

We also found the prevalence of AN to be high across age groups although we did observe a steady decrease in the prevalence of AN with increasing age. Formal recommendations of the American Diabetes Association (ADA) have recognised AN as a risk factor for T2DM in children and adolescents and have incorporated it into formal risk assessment protocols since 2000 [6]. Studies have found that approximately 60% to 92% of Black and Hispanic children with T2DM have AN [19] making it a highly sensitive marker in this age group. Indeed, in a recent survey among asymptomatic school-aged children screened for and confirmed with T2DM in Trinidad, all had AN [24]. The current study provides additional evidence of the value of AN as a valuable marker of T2DM risk in younger age groups. However, despite decreasing prevalence with age, more than one in three diabetic patients in this study over 70 years had AN: AN was still relatively prevalent in older age groups.

It is worth mentioning that this higher prevalence of AN in younger diabetic patients was partially explained by a higher prevalence of obesity in younger patients. However, even after adjusting for BMI and other factors, this trend of decreasing prevalence of AN with increasing age was still clearly observed. Litonjua et al. observed that newly diagnosed T2DM patients with AN “required markedly higher insulin doses to achieve euglycaemia” [15]. More severe insulin resistance in diabetic patients with AN may explain this decreasing prevalence of AN with age - a survivor effect.

The prevalence of AN was also high in both males (46%) and females (58%). Polycystic ovary syndrome (PCOS) is a well-established as an insulin-resistant state [25,26] and may have accounted for some or all of the higher prevalence of AN among women with T2DM.

To be a valuable adjunct to the T2DM screening process, AN should improve risk assessment by traditional risk factors. As recommended by the ADA, “the decision to test for diabetes should ultimately be based on clinical judgment and patient preference” [22]. Thus as an established risk factor for T2DM that is highly prevalent among diabetic patients in the Trinidadian population, the observation of AN alongside other risk factor(s) for T2DM should increase clinical suspicion of T2DM. Additionally, the incidental or intentional observation of this well-established skin marker of insulin resistance should prompt further risk assessment for T2DM.

For example, in this study approximately 60% of diabetic patients were found to be obese or overweight, and of these patients, 70% demonstrated AN. AN and obesity are associated with greater insulin resistance than obesity alone [27] and the finding of AN in an obese individual strengthens clinical suspicion of T2DM. Conversely, approximately 40% of diabetic patients were found to be of normal weight and as many as 30% of these demonstrated AN i.e. 12% of all diabetic patients were of normal weight yet manifested AN. AN in traditionally low-risk groups may be a valuable indicator of T2DM. However, in the absence of a control group in this study, we were unable to quantify this effect.

The interaction between BMI and ethnicity revealed interesting findings. Among obese diabetic patients, AN was most prevalent among East Indians. Among diabetic patients of normal weight however, Africans had a 1.7 times greater odds of demonstrating AN than East Indians of the same age, sex and waist circumference. Although the study was insufficiently powerful to show that this OR was different from one, this unexpected finding suggests that AN could be more prevalent among African than East Indian diabetic patients of normal weight, and this finding is worthy of further exploration in appropriately-powered studies.

AN can be rapidly assessed at the neck making it an extremely practical risk assessment tool. Examination for AN at other sites of the body can also marginally improve detection of T2DM. Among diabetic patients in this study, 52.7% had AN at the neck with or without AN at other sites, and a further 4.8% (approximately 1 in 20 diabetic patients) had AN only at other sites. Similar findings of neck involvement in 93% to 99% of AN was noted in the literature [13,21]. The low sensitivity of this further examination for AN at other sites, together with the additional time demands that it incurs, may render it impractical in routine practice. AN at other sites has also been less extensively described and quantified than AN at the neck and this may affect the validity of its routine assessment [13]. Potentially, the presence of AN at other sites may still provide useful evidence of T2DM risk in uncertain situations.

So far, we have discussed AN as a tool for improving the detection of undiagnosed T2DM. However, AN is also an important predictor of future T2DM. Stuart et al. described it as “a readily visible marker of endogenous hyperinsulinemia and thus, a marker for risk of developing non-insulin-dependent diabetes” [20]. Therefore, like other established risk factors for T2DM, the presence of AN is a valuable tool for recommendation of early lifestyle change for the prevention of T2DM and its complications, especially in a population where it is so readily manifest.

Apart from exploring AN as a valuable adjunct to T2DM screening, its link to other cardiovascular risk factors was explored. In this study, AN was found to have a demographic-adjusted association with each of obesity, hypertension and hypercholesterolemia. However, in our further multivariable analysis with all three variables (as well as demographic variables), only obesity remained associated with AN while the latter two did not. Consistent with this, a strong association between obesity, its accompanying insulin resistance, and AN is widely documented [8,19], and these findings emphasize that the presence of AN is more closely aligned with obesity than other cardiovascular risk factors.

One limitation of this study was that it did not allow for formal quantification of improvement in sensitivity of T2DM risk assessment attributable to the addition of AN to the pool of current risk factors. Such an assessment would provide valuable evidence to support health policy decision making. The promising results presented here favour further research into quantifying the benefit of AN in preparation for formal policy recommendation.

Another important limitation was that the study was conducted in a population of diabetic patients only. Therefore, the specificity of AN as a marker of T2DM could not be assessed. Study findings suggest that AN is a prevalent risk factor that can support clinical decision making. However, lack of information on the specificity of AN limits our ability to draw conclusions on whether the presence of AN alone or in traditionally low-risk groups is enough on its own to warrant screening for T2DM. If AN were found to be relatively non-specific, then its stand-alone value would be limited. Importantly, however, AN also predicts future T2DM making any measure of specificity difficult to interpret: while low specificity may mean that fewer cases will truly have T2DM, detection of AN provides an opportunity for preventing or delaying future T2DM. Schwartz reported that “it now appears that most, if not all, obesity-related AN, as well as syndromic AN, is related to insulin-resistant states” [8].

We also recognise that diabetic patients in our study were sampled only from tertiary hospitals and our findings may therefore be more representative of diabetic patients with more severe disease. Since AN is associated with more severe disease [15], our estimate of the prevalence of AN may be an over-estimation and should be extrapolated to the general population of diabetic patients with caution. Additionally, our inferences concerning the usefulness of AN in detecting undiagnosed T2DM, are based on the assumption that the distribution of risk factors for T2DM in already-diagnosed diabetic patients will be reflective of that seen in undiagnosed patients, and this assumption may not necessarily be true.

## Conclusions

Among diabetic patients in Trinidad, AN is highly prevalent both overall and across age, sex and ethnic groups. The high prevalence of this established risk factor for T2DM, together with the simplicity and convenience of its assessment, support the implementation of AN as a valuable addition to T2DM risk assessment in Trinidad.

We recommend that AN should be proactively observed in routine clinical practice in Trinidadian and similar ethnic settings and that a finding of AN should increase clinical suspicion of T2DM. Further study into the specificity of AN is needed in order to understand its value as a marker in traditionally low-risk groups.

## Abbreviations

AN: Acanthosis Nigricans; BMI: Body mass index; CI: Confidence interval; CVA: Cerebrovascular accident; IQR: Interquartile range; LRT: Likelihood ratio test; med: Median; MI: Myocardial infarction; OR: Odds ratio; RBG: Random blood glucose; SD: Standard deviation; T2DM: Type 2 diabetes mellitus.

## Competing interests

The authors declare that they have no competing interests.

## Authors’ contributions

ST and CM conceived the study. ST and CM designed and co-ordinated the study. SB and TS performed the statistical analyses. ST drafted the manuscript. SB, TS and ST revised the manuscript for important intellectual content. All authors read and approved the final manuscript.
